# 

**Published:** 1871-09

**Authors:** 


### INDEX OF SUBJECTS.

### ORIGINAL COMMUNICATIONS.

Rattle Snake's Poison, and its Remedies........................................457

Cholera Infantum........................................460

### EXTRACTS AND GLEANINGS.

Quinine, 463; The Sickness of Pregnancy: Its Cause and Treatment, 468—Treatment of Sore Nipple, 469; A New Mode of Administering Copaiva, 470; Carbuncles—Neuralgia Accompanying Shingles—Opium in Ulcerations, 471; Pertussis by Local Treatment—Alkaline in Skin Diseases—Voltile Liniment—Quinine Pills, 472; Emulsio Hydrocyanata—Velpeau's Mixture for Diarrhœa, Dysentery, etc.,—Treatment of Phoriasis by Balsam of Copiava, 473; Propylamin in Rheumatism—For Inflamed Nipples During Lactation, 474; Pulmonary Tuberculosis Treated by the Use of Cod Liver Oil, Saponified with Lime—For Dysentery—Phosphate of Lime in Sickness of Pregnancy—Anti-Hysteric Mixture—Carbolated Cerate Dressing in Varicose Ulcer of Leg, 475; Management of Measles—Wash in Phyalism—Sumac Poultice for Boils, 476; Permanent Contraction of a Limb Cured by Sub-Cutaneous Injection of Atropia—Anti rheumatic Liniment—Abortive Treatment of Felons—Peritonitis Meretricum, 477; Treatment of Infantile Paralysis—Ointment for Old Sores—Diaphoresis in Scarlatinal Dropsy, 478; A Substitute for the Compound Pill—Anti-Asthmatic Mixture—The Iodide of Potassium in the Treatment of Aneurism—Pyæmia and its Treatment, 479; Sesquichloride of Iron and Glycerine in Diphtheria and Croupous Affections—Anti diarrhœa Mixture—Bromide of Potassium an Antidote for Strychnine-Poisoning, 480; Treatment for Deafness, by Dr. Curtis—Imperial Mixture or Drink—Belladonna in Tonsilitis, 481; Carbuncles, 482; Arsenic in Prolapsus Uteri, from Enlarged Cirvix, 483; Carbolic Acid, 484; Sulphite of Soda in Carbuncle—Hooping-Cough—Formula for Gfving Quinine—Collin's Disinfecting Powder for Foul Apartments, 485; Treatment of Gonorrhœa—Chronic Ulcers—Quinine Biscuit—Management of Incontinence of Urine, 486; Gonorrhœal Peritonitis—Torticollis, New Method of Operating—To Prevent Infantile Gonorrhœal Ophthalia—Stricture of the Urethra, 487; Sulphite of Soda in Diabetes—A New Preparation of Pepsine—Tincture Erigeron Canadense in Hemorrhage, 488; Sulphites in Sypilis—Elixir Chloroform—Falling of the Hair—Capsicum in Asthma, 489; Medical Treatment of Ulcer of the Leg—A Good Prescription in Tuberculosis—Treatment of Bronchorrœa by Tar-Water, 490; Treatment of Buboes, etc.,—Good Prescription in Infantile Neuralgia, 491; Electricity in Neuralgia Tincture of Erigeron—Treatment of Syphilis—Ice in Affections of the Testicles—Sulphite Quinine in Erysipelas, 492; Treatment of Functual Indigestion, 493; Nitrate of Lead in Onychia—Sulpho-Carbolate of Zinc, 494; Muscular Rheumatism—Cholera Morbus—Ovarian—Neuralgia, 495; Permanganate of Potash—Hypoposphite of Potass. in chronic Bronchitis—Turpentine in Puerperal Prostration, 496; Sulpho-Carbolate of Sodium—Tonka Bean in Pertussis—Use of Arsenic, 497; Strychnia in Epilepsy—Subcutaneous Injection of Morphia in Dyspepsia—Sulphurous Acid in Chronic Vomiting, 498; Rice and its Use, 499.

### EDITORIALS, CORRESPONDENCE AND MISCELLANEOUS.

To our Subscribers—Our Associate and Corresponding Editors..........501
Character........................................502
Medical Society of Virginia........................................506
Correspondence........................................507
Editorial Correspondence........................................509
Letter from Mississippi........................................510
Truth is Mighty, etc........................................511
Books, Phamphlets, etc........................................512
Advertising Department........................................515

